# Cyclophosphamide for severe T-DXd-induced interstitial lung disease in low-HER2 breast cancer: a case report and mechanistic insights

**DOI:** 10.3389/fonc.2025.1638194

**Published:** 2025-10-27

**Authors:** Weichun Zhang, Xiaozhi Li, De Zeng, Wende Wang, Minna Chen, Wenwu Xue, Xiaofen Wen, Danxia Lin

**Affiliations:** The Department of Medical Oncology, the Cancer Hospital of Shantou University Medical College, Shantou, China

**Keywords:** trastuzumab deruxtecan, interstitial lung disease, cyclophosphamide, low-HER2 breast cancer, treatment

## Abstract

**Background:**

Trastuzumab deruxtecan (T-DXd) is an antibody-drug conjugate (ADC) that has shown significant efficacy in treating both HER2-positive and low-HER2 breast cancers. However, interstitial lung disease (ILD) remains a major adverse event associated with T-DXd treatment. Current management strategies for T-DXd-related ILD primarily rely on corticosteroids and immunoglobulins, with no established immunosuppressive regimen for steroid-refractory cases.

**Case description:**

A 49-year-old female with low-HER2 breast cancer developed Grade 4 ILD after receiving T-DXd treatment. She presented with severe respiratory symptoms, including chest tightness and hypoxia, and imaging revealed diffuse alveolar damage (DAD) pattern. Initial treatment with high-dose methylprednisolone and intravenous immunoglobulin showed limited improvement. Subsequently, low-dose cyclophosphamide (50 mg daily) was added, leading to rapid symptomatic and radiographic improvement. The patient’s condition stabilized, with significant reduction in lung inflammation, allowing for gradual tapering of corticosteroids and eventual discharge.

**Conclusions:**

This is the first reported case of successful cyclophosphamide treatment for Grade 4 T-DXd-induced ILD in a low-HER2 breast cancer patient with severe liver metastases. It highlights the potential efficacy of cyclophosphamide in treating severe T-DXd-induced ILD, particularly in steroid-refractory cases. The mechanism may involve its ability to inhibit macrophage proliferation and promote anti-inflammatory effects. Further prospective studies are needed to validate the role of cyclophosphamide in managing T-DXd-related ILD and to explore risk stratification for optimal toxicity management.

## Introduction

Trastuzumab deruxtecan (T-DXd) is an antibody-drug conjugate (ADC) composed of a humanized monoclonal antibody against human epidermal growth factor receptor 2 (HER2), a tetrapeptide-based cleavable linker, and a topoisomerase I inhibitor payload. T-DXd, studied extensively in the DESTINY-Breast series (1–4), has shown promising results in managing both HER2-positive and HER2-low-expressing breast cancers, significantly impacting the treatment landscape. However, interstitial lung disease (ILD) remains its most concerning adverse event, despite an overall manageable safety profile.

Powell et al. analyzed nine studies involving T-DXd (5), and the results showed that the overall incidence of ILD was 15.4% (grade 5, 2.2%), and most ILD patients experienced low-grade events (grade 1 or 2, 77.4%); 87.0% of patients had their first ILD within 12 months of starting treatment, with a median of 5.4 months (range <0.1-46.8 months). The median time to onset of grade 5 was 3.2 months (range <0.1-20.8 months). In addition, in most cases, low- grade ILD (grade <= 2) can be effectively treated, but in some cases it may be fatal. This is due to the non-specificity of ILD symptoms, signs and imaging, leading to untimely clinical diagnosis, and ultimately delayed treatment, which is life-threatening in severe cases. Therefore, monitoring and management of ILD is an important part of T-DXd treatment (6). The severity of T-DXd-ILD appears dose-dependent and associated with cytotoxic payload in alveolar macrophages. The cornerstone of T-DXd-ILD management is corticosteroid therapy, with dose adjustments based on adverse event severity. If no improvement occurs within 5 days, additional immunosuppressants (e.g., infliximab, IVIG, mycophenolate mofetil) should be considered. Currently, there is no established immunosuppressive regimen for steroid-refractory T-DXd-ILD.

## Case description

A 49-year-old female was diagnosed with left breast invasive ductal carcinoma (cT2N0M0, Luminal B subtype, HER2-negative) in June 2022. She received neoadjuvant chemotherapy from July to December 2022, consisting of 4 cycles of docetaxel followed by 4 cycles of epirubicin and cyclophosphamide. On January 5, 2023, she underwent axillary lymph node dissection, unilateral mastectomy, sentinel lymph node biopsy, and nanocarbon lymph node tracing. Postoperative pathology revealed ypT2N1aM0, HER2 2+ (FISH-negative). She completed adjuvant radiotherapy from February to March 2023, targeting the chest wall and regional lymph nodes. From February to April 2023, she received endocrine therapy with abemaciclib and exemestane, experiencing grade I diarrhea during treatment.

**Figure 1:**
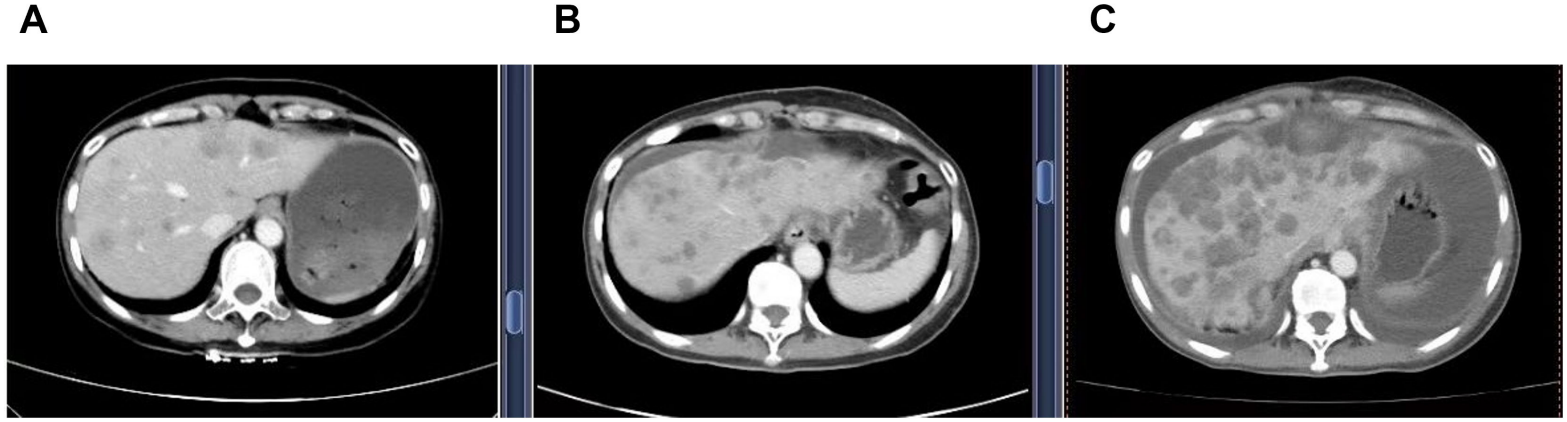
Serial contrast-enhanced abdominal CT images demonstrating progression of hepatic metastases. **(A)** (21 April 2023) Multiple new hypodense liver nodules of varying sizes, the largest measuring 5.0 x 3.6 cm. The lesions demonstrate mild, heterogeneous, enhancement with ill-defined margins. These findings are new compared to the prior examination and are consistent with metastatic disease. **(B)** (16 July 2024) Increase of low-density hepatic nodules with ill-defined margins, and mild heterogeneous enhancement. The dominant lesion in the quadratic lobe measures 5.7 cm x 4.9 cm, showing peripheral fusion and subcapsular extension, consistent with disease'progression. **(C)** (22 October 2024) Increase in both size and number of multifocal low-density hepatic nodules, with mild heterogeneous enhancement, ill-defined margins, and areas of confluence. The largest lesion in the left lobe measures 7.1 cm x 4.9 cm. These findings are consistent with progressive metastatic disease.

The patient was treated on multiple occasions between April 2023 and November 2024 for progression of liver metastases. The treatment details are summarized in **Table 1** and **Figure 1**.

**Table 1:** Timeline of patient diagnosis and treatment (see also **Figures 1A–C**).

Time Period	Evidence of Progression	Treatment Regimen and Cycles	Adverse Events
Apr 2023 - Aug 2023	New hepatic metastases identified (FIGURE 1A)	Anlotinib + Disitamab Vedotin for 9 cycles	None reported
Sep 2023 - Jan 2024	Mixed response in hepatic metastases	D-TACE (3 cycles) + TAI (2 cycles)	Severe thrombocytopenia
Apr 2024 - Jun 2024	Enlargement of hepatic metastases & new retroperitoneal LN metastasis	Disitamab Vedotin + Anlotinib for 3 cycles	None reported
Jul 2024 - Aug 2024	Progression of hepatic metastases (FIGURE 1B)	Utidelone + Capecitabine for 2 cycles	Grade II leukopenia & Grade II oral mucositis
Oct 2024 - Nov 2024	Increased size/number of hepatic metastases (FIGURE 1C)	T-DXd for 2 cycles	ILD

LN, Lymph Node; D-TACE, Drug-Eluting Beads Transarterial Chemoembolization; TAI, Transarterial Infusion; T-DXd, Trastuzumab Deruxtecan; ILD, Interstitial Lung Disease.

**Figure 2:**
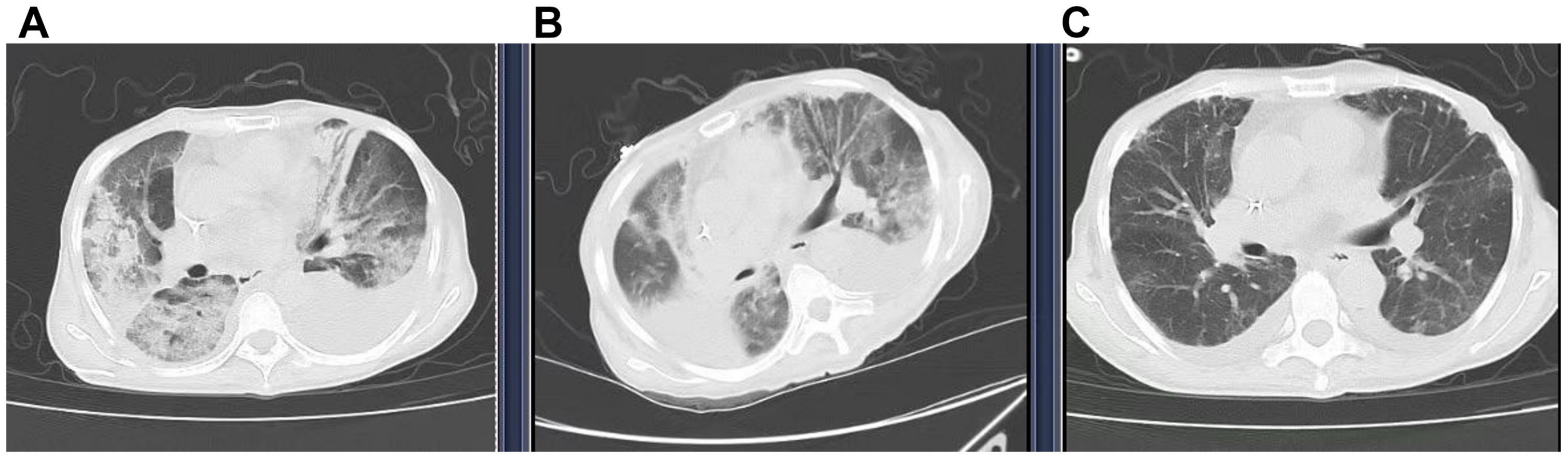
Computed tomography (CT) showed the transition of ILD. **(A)** (29 Nov 2024) New diffuse bilateral patchy opacities with heterogeneous attenuation and ill-defined margins. Increased bilateral pleural effusions (moderate left, small right). **(B)** (5 Dec 2024) Diffuse bilateral opacities with heterogeneous attenuation, partial resolution (RML, left lung) but progression (RUL, right upper lobe). The right pleural effusion has increased and is partially encapsulated, whereas the left effusion has decreased slightly. **(C)** (11 Dec 2024) Significant resolution of bilateral diffuse opacities and reduction of bilateral pleural effusions.

On November 28, 2024, she experienced sudden chest tightness and shortness of breath, with peripheral blood oxygen saturation (SpO_2_) of 50-70% on room air. Fine moist rales were auscultated in the upper and middle lung fields. Bedside ultrasound showed large bilateral pleural effusions, atelectasis, normal cardiac systolic function, and no signs of thrombosis. No significant abnormalities were found on urgent laboratory testing, including procalcitonin, D-dimer, NT-proBNP, and troponin T levels (**Table 2**). High-resolution chest CT (≤1.5mm thick, **Figure 2A**) showed diffuse patchy lesions in both lungs with uneven density and unclear edges, and bilateral pleural effusions (moderate on the left, small on the right. Based on the exclusion of alternative diagnoses (infection, pulmonary embolism, and heart failure) through the above workup, along with a history of T-DXd exposure, characteristic clinical features, and supportive CT imaging, a definitive diagnosis of interstitial pneumonitis was made.

**Table 2:** Laboratory parameters of the patient.

Date	Test items	Results	Reference range	Unit	Testing methods
November 29, 2024	High-sensitivity troponin (TNT-hs)	15.3	0-14.0	pg/ml	Chemiluminescence
November 29, 2024	N-terminal pro-B-type natriuretic peptide (pro-BNP)	116	<125	pg/ml	Chemiluminescence
November 29, 2024	Myoglobin (MYO)	36.5	25-58	pg/ml	Chemiluminescence
November 29, 2024	Procalcitonin (PCT)	0.627	0-0.05	ng/ml	Chemiluminescence
November 29, 2024	Interleukin-6 (IL-6)	10.1	0-7	pg/ml	Chemiluminescence
November 29, 2024	D-dimer (DDHS)	4792	0-550	ng/ml	Chemiluminescence
November 29, 2024	Salivary glycan antigen (KL-6)	2155	0-500	U/ml	Chemiluminescence
December 9, 2024	Aspergillus antigen (galactomannan)	0.18	0-0.49		Enzyme-Linked Immunosorbent Assay

A bedside right thoracentesis was performed to drain pleural effusion, and symptoms improved slightly after intravenous infusion of 80 mg methylprednisolone. Oxygen supplementation via a non-rebreather mask at 15 L/min stabilized her SpO_2_ between 94% and 99%. However, despite receiving a three-day course of methylprednisolone (80 mg q12h, from November 29 to December 1, 2024) for anti-inflammatory purposes, the patient continued to experience recurrent chest tightness and decreased SpO_2_. Then she was referred to our department with Grade 4 ILD (Diffuse Alveolar Damage/DAD pattern on CT) and life-threatening respiratory failure (NCI-CTCAE v5.0). Accordingly, the treatment was escalated to high-dose methylprednisolone (250 mg every 12 hours for 4 days, from December 2 to December 5) and intravenous immunoglobulin (20g daily for 3 days, totaling 1g/kg, from December 2 to December 4). To manage the left pleural effusion, a therapeutic thoracentesis was performed. Following three days of high-dose glucocorticoid and intravenous immunoglobulin pulse therapy, the patient maintained stable peripheral oxygen saturation (100%) on 10 L/min non-rebreather mask. However, repeat chest CT (5 December 2024, **Figure 2B**) demonstrated diffuse patchy opacities in both lungs with heterogeneous attenuation and ill-defined margins. Comparative assessment revealed partial resolution in the right middle lobe and left lung, but mild progression in the right upper lobe. Methylprednisolone was tapered to 55 mg q12h, and low-dose cyclophosphamide (50 mg qd) was added. Following six days of low-dose cyclophosphamide combination therapy, the patient demonstrated marked symptomatic improvement with resolution of chest tightness, maintained SpO_2_ of 100% on 4 L/min nasal cannula, and improved mobility in bed. A repeat chest CT (11 December 2024, **Figure 2C**) revealed bilateral diffuse patchy opacities with heterogeneous density and ill-defined margins, which had significantly decreased in size compared to prior imaging. Methylprednisolone was further reduced to 45 mg q12h. On December 13, the patient began oral prednisone with a slow dose reduction. She was discharged on December 16 with stable vital signs and instructions for outpatient follow-up. Following discharge, the patient continued oral cyclophosphamide at a constant dose and prednisone (with a slow tapering regimen starting at 35 mg twice daily and reduced by 20 mg per week) until both medications were discontinued on January 10, 2025, but declined serial complete blood count monitoring and prophylactic trimethoprim-sulfamethoxazole (TMP-SMX) for Pneumocystis jirovecii pneumonia (PJP) prevention. At 30-day follow-up, she maintained an oxygen saturation of 100% on 2 L/min nasal cannula; however, she exhibited marked cachexia, suggesting possible progression of her underlying malignancy. The specifics of the treatment protocol, including dosage and duration, are summarized in **Table 3**.

**Table 3:** Management and outcomes of T-DXd-induced interstitial lung disease.

Date	Methylprednisolone (mg/q12h)	Prednisolone (mg bid)	Immunoglobulin (g/d)	Cyclophosphamide (mg/d)	Radiological response on chest CT
29 Nov – 1 Dec 2024	80				FIGURE 2A(29 Nov 2024)
2 Dec – 4 Dec 2024	250		20		FIGURE 2B(5 Dec 2024)
December 5, 2024	250			50	
6 Dec – 9 Dec 2024	55			50	
10 Dec – 12 Dec 2024	45			50	FIGURE 2C(11 Dec 2024)
13 Dec – 19 Dec 2024		35		50	
20 Dec – 26 Dec 2024		25		50	
27 Dec 2024 – 2 Jan 2025		15		50	
3 Jan – 9 Jan 2025		5		50	

## Discussion

The diagnosis of drug-induced interstitial lung disease (DI-ILD) is one of exclusion (7), as its symptoms (e.g., cough, dyspnea, fever, and decreased SpO_2_) are nonspecific. An accurate diagnosis requires a thorough assessment that rules out other causes of lung injury, such as infections, heart failure, radiation-induced lung injury, and pulmonary embolism. This evaluation is based on integrating findings from clinical history (including medications and radiation exposure), imaging, pulmonary function tests, and laboratory results (8). Several clinical studies (9) suggest that serum biomarkers, notably Salivary glycan antigen (KL-6) and surfactant protein-D (SP-D), hold potential as tools for the early detection of T-DXd-induced ILD; however, their clinical applicability requires further validation in larger, prospective cohorts.

High-resolution chest CT (≤1.5 mm thick) is the preferred tool for diagnosing ILD, with common patterns including diffuse alveolar damage (DAD), nonspecific interstitial pneumonia (NSIP), hypersensitivity pneumonitis (HP), and organizing pneumonia (OP) (10, 11). In T-DXd-related ILD, OP and HP patterns are predominant, while DAD is usually present in grade 4 DI-ILD, characterized by diffuse or multifocal ground-glass opacities and consolidation, often with traction bronchiectasis. Tomohisa Baba’s report indicates that ILD with a DAD pattern, which typically responds poorly to corticosteroids, had a fatal outcome in 42.1% (8/19) of cases even with immediate high-dose corticosteroid therapy after onset (10). F. M. Costa reported a case of grade 4 T-DXd–induced ILD with DAD pattern (12). The patient received pulse methylprednisolone therapy (0.5 g/day for 3 days), followed by maintenance dosing (1 mg/kg/day). After 7 days of corticosteroid treatment without improvement, indicating steroid-refractory disease, infliximab was initiated. However, no improvement in High-Flow Nasal Cannula (HFNC) parameters was observed by day 13, with concomitant radiographic progression.

Because of the risk of rapid ILD progression, careful management of T-DXd–related pulmonary toxicity is essential (8). Symptomatic ILD that does not interfere with activities of daily living (grade 2) warrants permanent discontinuation of T-DXd and prompt initiation of systemic corticosteroids. For grade 3 or higher ILD, high-dose methylprednisolone (e.g., 500–1000 mg/day for 3 days) should be initiated immediately, followed by prednisolone (or equivalent) at ≥1 mg/kg/day for at least 14 days, and then tapered gradually over ≥4 weeks. If clinical or radiographic worsening occurs, or there is no improvement (especially within 5 days), additional immunosuppressants (e.g., mycophenolate mofetil and azathioprine) and/or local standard management should be considered.

This is the first reported case of severe (Grade 4) T-DXd-induced ILD in a low-HER2 breast cancer patient successfully treated with cyclophosphamide. Current management of T-DXd-related ILD relies on glucocorticoids and immunoglobulin, without established immunosuppressive regimens. Notably, cyclophosphamide led to rapid symptomatic (chest tightness) and radiographic improvement in this case. It suggests cyclophosphamide may bridge the gap in steroid-refractory cases, but requires validation in prospective studies.

The management of severe, steroid-refractory T-DXd-ILD (NCI grade ≥3) with a DAD pattern on CT remains challenging and is associated with high mortality. Based on previous successful experiences at the Second and Third Affiliated Hospitals of Sun Yat-sen University using low-dose cyclophosphamide for steroid-refractory T-DXd-ILD, we added low-dose cyclophosphamide to the regimen after confirming steroid resistance. This intervention resulted in significant clinical improvement. Compared to mycophenolate mofetil or azathioprine (which are mentioned in T-DXd-ILD management guidelines), low-dose cyclophosphamide may offer a superior risk-benefit profile in this setting, owing to its potentially lower myelosuppressive toxicity at controlled doses. This approach is further supported by a retrospective study by H. Katahara et al., which indicated the efficacy of cyclophosphamide in corticosteroid-refractory ILD, particularly cases induced by molecularly targeted therapies (13). Notably, a patient in that series (Case 9), whose condition was similar to ours, also showed improvement in respiratory function and imaging following cyclophosphamide treatment.

Recent studies have shown a correlation between T-DXd-related ILD and alveolar macrophages(AMs) uptake of T-DXd (14, 15). The potential mechanism involves the binding interaction between the Fc fragment of trastuzumab and the Fcγ receptor on AMs, which facilitates the uptake and effector function of T-DXd. Further experiments showed that deglycosylation of T-DXd’s N-glycan can reduce the incidence and severity of ADC-related ILD (15). This finding provides valuable insights for optimizing ADC design and treatment strategies to mitigate ILD risk. In multiple *in vivo* and *in vitro* studies, cyclophosphamide has been shown to significantly suppress macrophage proliferation and phagocytic activity (16) while promoting polarization toward an anti-inflammatory (M2) phenotype, thereby exerting anti-inflammatory effects (17). This mechanism may underlie cyclophosphamide’s efficacy in treating T-DXd-related ILD.

In clinical practice, physicians need to take into account the patient’s condition, treatment history, and individual characteristics, and closely monitor the patient’s pulmonary symptoms and imaging changes to promptly detect and manage T-DXd–related ILD, thereby enhancing treatment safety and efficacy. Therefore, it is important to thoroughly evaluate individual risk factors in all T-DXd users. Some studies have analyzed the risk factors for T-DXd–related ILD and identified several potential factors (5, 18, 19), including age, Japanese ethnicity, T-DXd dose, SpO_2_ levels, moderate or severe baseline renal insufficiency (based on the Cockcroft-Gault formula), presence of pulmonary comorbidities (excluding lung cancer and pulmonary metastases), prior use of immune checkpoint inhibitors, and time since initial diagnosis. Some evidence also suggests that a history of thoracic radiotherapy may contribute to increased risk (20). In addition, the patient in this case had severe liver metastasis, which suggests a potential mechanism for increased risk of T-DXd–induced interstitial lung disease, whereby hepatic dysfunction may cause systemic drug accumulation and promote a pro-inflammatory, immunodysregulated state, collectively exacerbating pulmonary injury. However, large-scale prospective clinical studies are currently lacking to clarify the strength and mechanism of this association.

## Conclusion

This is the first reported case of successful cyclophosphamide treatment for Grade 4 T-DXd-induced ILD in a low-HER2 breast cancer patient with severe liver metastases. Our findings suggest that cyclophosphamide may be an effective therapeutic option, potentially through its ability to inhibit macrophage proliferation and promote anti-inflammatory polarization. This observation warrants further investigation into its role in T-DXd-related ILD. Additionally, this case highlights the importance of assessing comorbidities (e.g., liver metastases) for optimal toxicity management. Future studies should explore risk stratification and tailored strategies to balance ILD prevention with T-DXd efficacy.

## Data Availability

The original contributions presented in the study are included in the article/supplementary material. Further inquiries can be directed to the corresponding author.
